# Current Novel Advances in Bronchoscopy

**DOI:** 10.3389/fsurg.2020.596925

**Published:** 2020-11-16

**Authors:** Jeffrey Jiang, Stephanie H. Chang, Amie J. Kent, Travis C. Geraci, Robert J. Cerfolio

**Affiliations:** Department of Cardiothoracic Surgery, New York University Langone Health, New York, NY, United States

**Keywords:** Intuitive Surgical, bronchoscope, electromagnetic navigation bronchoscopy, robotic bronchoscopy, ion entydoluminal, monarch

## Abstract

Screening for lung cancer has changed substantially in the past decade since The National Lung Screening Trial. The resultant increased discovery of incidental pulmonary nodules has led to a growth in the number of lesions requiring tissue diagnosis. Bronchoscopy is one main modality used to sample lesions, but peripheral lesions remain challenging for bronchoscopic biopsy. Alternatives have included transthoracic biopsy or operative biopsy, which are more invasive and have a higher morbidity than bronchoscopy. In hopes of developing less invasive diagnostic techniques, technologies have come to assist the bronchoscopist in reaching the outer edges of the lung. Navigational bronchoscopy is able to virtually map the lung and direct the biopsy needle where the scope cannot reach. Robotic bronchoscopy platforms have been developed to provide stability and smaller optics to drive deeper into the bronchial tree. While these new systems have not yet proven better outcomes, they may reduce the need for invasive procedures and be valuable armamentarium in diagnosing and treating lung nodules, especially in the periphery.

## Introduction

Screening for lung cancer has changed substantially in the past decade. The National Lung Screening Trial demonstrated significant utility for low dose computed tomography (CT) scans in patients with high risk profiles by increasing early detection of lung cancer with decreased mortality (1, 2). With the advent of screening, 1.6 million new pulmonary nodules are detected annually, posing diagnostic dilemmas in evaluating these lesions (3). While many of these nodules are small and can be monitored with serial imaging, many require tissue for diagnosis and eventual treatment. The number of invasive diagnostic procedures has subsequently increased in kind.

Prevailing modalities for obtaining tissue diagnosis of pulmonary nodules include transthoracic image guided biopsy and endoscopic bronchoscopy and ultrasound (EBUS). Both have limitations. Transthoracic biopsy is largely useful for peripheral, small lesions, and has higher yield than bronchoscopy, but is inadequate for sampling central lesions and mediastinal lymph nodes (4). There is a substantial risk of lung injury and iatrogenic pneumothorax, which is increased in patients with emphysematous changes (5), resulting in a reluctance for transthoracic biopsy for lesions close to major vascular structures or in bullous lungs. Endoscopic bronchoscopy is often still required staging in malignant lesions. EBUS remains one standard method of tissue diagnosis, as the modality allows for staging mediastinal lymph nodes and evaluating endobronchial involvement. However, it is limited to central and large tumors, with reported of low diagnostic yield for other nodules. Surgical biopsy remains as the final option, especially for peripheral lesions, but is an invasive procedure. Additionally, surgical resection may require preoperative marking for small nodules or those not directly on the pleural surface.

## History of Bronchoscopy

Bronchoscopy has undergone a number of iterative improvements to become a useful and versatile diagnostic tool. Direct bronchoscopy originated as a tool for retrieval of foreign objects, and evolved from the laryngoscope used by otolaryngologists. Flexible bronchoscopy was introduced by Dr. Ikeda, a thoracic surgeon at the National Cancer Center in Japan, after applying the fiberoptic imaging used by endoscopists to a smaller channel. Biopsy forceps were easily adapted, and trans-bronchial fine need aspiration for cytology quickly followed (6). Miniaturized ultrasound probes, first with a radial probe and then the convex probe, soon made bronchoscopy the standard of care in staging the mediastinum (6–8). While the advancements in bronchoscopy have allowed it to move from direct line of sight to endobronchial, and then to transbronchial biopsy, more peripheral pulmonary lesions remain a challenge.

## Electromagnetic Navigational Bronchoscopy

Given the risks of transthoracic approaches and invasiveness of surgical biopsy, recent advancements have been developed regarding image guidance to extend the bronchoscope's reach. Electromagnetic navigational bronchoscopy (ENB) relies on high resolution CT and an electromagnetic (EM) field generated around the patient's chest. CT images are reconstructed into a three-dimensional map and loaded to generate a virtual bronchoscopist's view. A steerable probe that can be sensed by the field is loaded into the tip of a flexible bronchoscope and select known points in the tracheobronchial tree are mapped to the virtual lungs to synchronize the images to the EM field. The probe, along with an extendable working channel, can then be advanced past the scope tip into smaller bronchi and drive along the virtual bronchial map to reach the target (9).

While some reports indicate ENB is safe and has allowed for better sample yield of peripheral lesions, the technique is highly operator and anatomy dependent. Upper and middle lobe lesions, large lesions >2 cm, a bronchus sign (imaging of a bronchus leading to the lesion) and concurrent use with radial EBUS have been shown to improve yield (10, 11). Meta-analysis of 39 studies indicate pooled diagnostic yields around 70%, although with wide variability and a risk of 1–2% pneumothorax (10) (Table 1). A more recent pool of 16 studies demonstrates a similar combined yield of 64.9% and sensitivity to detect malignancy of 71%, with a 3% pneumothorax rate and 1.6% tube thoracostomy rate (11). Registry data review of a variety of centers show worse diagnostic utility with ENB (yield of 38.5%) compared to radial EBUS (yield of 57%), raising the concern that efficacy may not translate from specialized centers to the community (12). Comparison of ENB to CT guided transthoracic biopsy has indicated that the diagnostic yield of bronchoscopy is still lacking. A single center retrospective review of 285 patients undergoing ENB or CT guided biopsy demonstrated yields of 66 vs. 86%, respectively. Sufficient yield for molecular analysis was similar between both modalities (89 vs. 82%, respectively). Complication rates were significantly higher complications in transthoracic biopsies compared to CT guided biopsy with increased incidence of pneumothorax (29 vs. 4%) and bleeding (17 vs. 3.3%), though thoracostomy tube placement and significant bleeding rates were similarly low (13). While ultimate interventions and major complications remained low, the higher rate of bleeding and pneumothorax requires admission and observation to ensure serious sequelae do not develop.

**Table 1:** ENB Clinical Data.

**Trial**	**Study type**	**Localization**	**Diagnostic yield**	**Complications**
Wang et al. (10)	Meta-Analysis	–	46–86.2%	1.5% pneumothorax
	*n* = 39		Pooled 70%	0.6% tube thoracostomy
Gex et al. (11)	Meta-Analysis	97.4%	55.7–87.5%	3.1% pneumothorax
	*N* = 15		Pooled 65.9%	1.6% tube thoracostomy
				0.9% bleeding
Ost et al. (12)	Registry	–	Bronchoscopy 53.7%	1.7% pneumothorax
AQuIRE	15 centers, *n* = 581		rEBUS 57%	0.2% bleeding
			ENB 38.5%	
			rEBUS+ENB 47.1%	
Bhatt et al. (13)	Cohort *n* = 285		ENB 66%	Pneumothorax (tube)
	ENB vs. Transthoracic		TTB 86%	ENB 4% (2.7%)
	150, 150 procedures			TTB 29% (1.3%)
				Bleeding (symptomatic)
				ENB 3.3% (2%)
				TTB 17% (1.3%)

ENB has been applied very successfully in the operating room for locating lesions for resection, especially during robotic operations. Without the ability to palpate for masses, robotic surgeons often rely on visual cues of mass location and can be aided by tattoo. ENB can help locate peripheral nodules for indocyanine green injection for precise resection. In our experience of 93 patients undergoing segmentectomy, ENB was able to locate 86% of lesions with no ENB related complications (14). Data of ENB is summarized in Table 1.

Technical concerns exist primarily around stability and extension of the probe/catheter complex past the bronchoscope. Catheter slippage can occur, especially when significant torque is necessary to create a stable position and during tool exchanges. Visualization at the distal subsegmental bronchi is also no longer real-time and relies on the virtual image after the probe is extended past the bronchoscope, which can make navigating sharply angulated, small bronchi difficult. Despite the technical difficulties, ENB is the most commonly used method to reach the peripheral bronchial tree for tissue sampling and remains the primary alternative to transthoracic biopsy with a more favorable risk profile, albeit with lower diagnostic yield. Tagging nodules endobronchially is also beneficial during sub-lobar resections and can locate nodules when unable to palpate or obscured by lung parenchyma (14).

## Robotic Bronchoscopy Platforms

The difficulties of ENB and suboptimal yield of traditional bronchoscopy has led to the development of robotic bronchoscopy. The robotic platform uses a similar virtual map generated from reconstructed high-resolution CT and EM field mapping, but has redesigned the bronchoscope and utilizes robotic arms to maneuver and drive it forward. Two robotic platforms are currently commercially available: Monarch™ (MA; Auris Health, Redwood City, CA), FDA approved in March 2018; and the Ion™ Endoluminal Platform (IEP; Intuitive, Sunnyvale, CA) that became FDA approved in February 2019.

The two platforms consist of largely similar equipment including a cart with robotic arms, the bronchoscope, the tower, and a controller. The Monarch™ system's bronchoscope consists of an 130° articulating sheath and an inner bronchoscope that telescopes out of the sheath and can flex 180° in any direction. All part of the scopes can be positionally parked for stability during tool exchanges and biopsy. The controller is modeled after current generation game controllers with two joysticks and minimal buttons (15). The Ion™ Endoluminal Platform uses a single bronchoscope/catheter complex and robotic arm. The scope consists of a catheter measuring 3.5 mm outer diameter and 2 mm working channel and a vision probe that loads into the working channel. The catheter includes fiber optic shape sensors that provide real time precise location and catheter shape information throughout the navigation and biopsy process and allows it to park the length of the catheter in its current formation for stability. The vision probe requires extraction once navigation is complete and biopsy is done under virtual guidance. Existing technologies, including radial EBUS, fluoroscopy and navigational bronchoscopy are integrated into both tower systems (16).

Early studies have been promising. An initial feasibility and safety study by Rojas-Solano et al. has shown a good safety profile, albeit in a small cohort. Fifteen patients with peripheral lesions and a bronchus sign underwent robotic bronchoscopy with the Monarch™ system and 93% of targets were able to be biopsied. Average tumor size was 26 mm. One patient required conversion to conventional bronchoscopy as the robotic parameters were set incorrectly. Another patient was non-diagnostic and subsequently underwent surgical biopsy for diagnosis of malignancy. No patients suffered pneumothorax or bleeding. Early procedure times had a median of 45 min, which dropped by more than half by the end of the series (17). A multicenter prospective study of 46 patients demonstrated similar results with successful navigation and biopsy in 95.6% of patients confirmed by radial EBUS. There was one pneumothorax (4.3%) requiring a tube thoracostomy (Table 2A). Yield and diagnosis are pending (18). A recent retrospective multicenter study in 165 patients with 167 lesions showed an 88.6% navigation rate when confirmed by radial EBUS and a conservative diagnostic yield of 69% and a maximum of 77%. Mean lesion size was 25 mm with 71% under 30 mm and 63.5% demonstrating a pre-procedure bronchus sign. In lesions where an eccentric view on radial EBUS was seen, diagnostic yield was 71%, higher than reported with radial EBUS alone. Complications included a 3.6% rate of pneumothorax and 2.4% rate of bleeding, comparable to other bronchoscopy trials (19). Two prospective single-arm multicenter trials, the BENEFIT and TARGET trial, are ongoing. Preliminary data from the BENEFIT trial demonstrate 96% localization rates and similarly low complication rates. TARGET is currently enrolling (21, 22).

**Table 2A:** Robotic bronchoscopy clinical trial data.

**Trial**	***n***	**Localization**	**Diagnostic yield**	**Complications**
**Monarch**™ **platform**				
Rojas-Solano et al. (17)	15	96% (one conversion to conventional)	- (1 required surgical biopsy)	0%
Chen et al. (18)	46	95.6%	-	4.3% pneumothorax, 2.1% tube thoracostomy
Chaddha et al. (19)	167	88.6%	69–77%	3.6% pneumothorax2.4% tube thoracostomy2.4% airway bleeding
**Ion**™ **endoluminal platform**				
Fielding et al. (20)	29	96.6%	79.3%	0%

One study has published data of the Ion™ system in 29 patients with intriguing yield data and an acceptable safety profile. Average tumor size was 12 mm with 96.6% localization and tissue sampling success. Diagnostic yield was 79.3 and 88% were malignant. Bronchus sign was present in 58.6% of all biopsied lesions. Procedure times, however, were fairly long, initially averaging 95 min before dropping to 61 min. The authors reported no complications (20). Our institution has used the Ion platform for 9 patients with a single surgeon immediately prior to resection for preoperative tattooing (Table 2B). Tattooing is done to help identify nodules for potential sub-lobar resection as they can difficult to visualize and cannot be felt on the robotic platform. Of our series, seven patients had successful navigation and dye injection. Two were converted to ENB and successfully tattooed. Mean duration of bronchoscopy was 13 min and none had complications related to bronchoscopy. Average length of stay was 1 day. The PRECIsE trial is a prospective single arm multicenter trial currently enrolling for the Ion™ Endoluminal system (21, 23).

**Table 2B:** NYU robotic clinical data with Ion™ platform.

**Baseline characteristics**		**Procedure details and outcomes**	
N = 9		Length of Bronchoscopy	13 ± 6 min
Age	69 ± 7.4	Successful Tattoo	7 (78%)
Male	4	R0 resection	9 (100%)
FEV1	90 ± 20.7	Complications	0 (0%)
DLCO	76 ± 17.6	Length of Stay	1 ± 0.3
Stage	1A (8), IIA (1)		
COPD	1 (11%)		
Smoker	6 (67%)		

## Discussion

Electromagnetic navigational bronchoscopy and robotic bronchoscopy have both expanded the reach of conventional bronchoscopy and EBUS. Virtual pathfinding and navigation have allowed the working channel to extend past what the camera can see and fit through. The ENB system has allowed CT imaging to not just guide operative planning, but be a real time GPS for sampling and marking peripheral lesions for diagnosis and surgical resection. While this has been an important step forward in advancing endobronchial therapies, operator dependence and technical prowess factor into the debate over ENB's overall usefulness in boosting diagnostic yield. The benefits of robotic assisted platforms largely stem from a retooling of the bronchoscope into one with precise movements, adjustable angulation, and increased stability. Reliable sampling of peripheral lesions necessitates the ability to navigate to a target and remain in stable position while instruments and needles are exchanged. Robotic assistance increases dexterity to make subtle or acute changes in navigation. The increased structural support of the sheath and scope, as well as the fiberoptic shape sensing, allows for more leverage when making complex turns, and aids in positional parking. Continuous visualization of the peripheral airways, with one platform also offering direct visualization of biopsy tools, allows for more accurate biopsy deployment. These attributes would seem to make robotic bronchoscopic navigation and biopsy safer and extend the reach compared to conventional bronchoscopy.

Current data is still ongoing to confirm if these technical advantages translate to an improved clinical experience for the patient. However, the reports published are promising and have good safety profiles. If improved diagnostic yield pans out, patients may be spared higher risk transthoracic biopsy and multiple staging procedures. In addition, with reliable navigation, robotic assisted platforms may be utilized for perioperative marking, whether through fiducial placement or tattoo, and obviate a separate marking procedure by CT guidance. For non-operative patients, the robotic platform may be a stable, accurate avenue for delivering endoluminal therapies to all corners of the lung.

All reports of robotic assisted bronchoscopy are from the past couple of years, and adoption of the platform remains in its infancy. Drawbacks to the technology include cost, increased complexity in the operating room, increased procedure time, and a learning curve without a proven benefit. Further studies are needed to evaluate the efficacy of robotic bronchoscopy. All early data needs to be evaluated with the knowledge that cost, procedure time, and efficacy improve with increasing experience due to the learning curve associated with any novel technology. Thoracic robotic surgery initially was considered inefficient due to cost, operating time, and lack of benefit over traditional minimally invasive platforms. However, persistence, practice and patience has demonstrated the benefit of the robotic platform for thoracic surgery. With refinement and familiarity, robotic assisted bronchoscopy may similarly become an essential step forward in the diagnosis and treatment of peripheral pulmonary nodules.

## Author Contributions

JJ: writing, research, and editing. SC, AK, TG, and RC: writing and editing. All authors contributed to the article and approved the submitted version.

## Conflict of Interest

RC discloses past relationships with AstraZeneca, Bard Davol, Bovie Medical Corporation, C- SATS, ConMed, Covidien/Medtronic, Ethicon, Fruit Street Health, Google/Verb Surgical, Intuitive Surgical, KCI/Acelity, Myriad Genetics, Neomend, Pinnacle Biologics, ROLO-7, Tego, and TransEnterix. The remaining authors declare that the research was conducted in the absence of any commercial or financial relationships that could be construed as a potential conflict of interest.
